# Prevalence of *Campylobacter* spp. in Poultry in Three Spanish Farms, A Slaughterhouse and A Further Processing Plant

**DOI:** 10.3390/foods8030111

**Published:** 2019-03-26

**Authors:** Iratxe Perez-Arnedo, Elena Gonzalez-Fandos

**Affiliations:** Food Technology Department, CIVA Research Center, University of La Rioja, 26006 La Rioja, Spain; ipearn@gmail.com

**Keywords:** meat safety, *Campylobacter*, poultry, foodborne pathogens

## Abstract

The present study was conducted to investigate the prevalence of *Campylobacter* spp. in a selection of poultry flocks and the corresponding broiler carcasses as well as the possible impact of contamination during slaughter and processing. Samples of the same flock at different ages in three farms (A, B and C) were taken for the determination of *Campylobacter* spp. The same broiler flocks were examined at different stages of one slaughterhouse and at a further processing plant. The slaughterhouse environment and processing equipment were sampled. *Campylobacter* spp. was not detected in 7 and 14-day-old broilers in any of the three farms studied. However, *Campylobacter* spp. was detected in 35 and 42-day-old broilers at two farms (Farm A and B). This pathogen was detected in both dirty and clean transport crates, in scalding water, and on the defeathering machine and the working table at the end of the working day, but not at the beginning. After defeathering, *Campylobacter* spp. was detected in all of the sampled carcasses. *Campylobacter* spp. was detected in all of the carcasses and the poultry meat portion samples from Farm C, although it was not detected at the farm level. This suggests that *Campylobacter* spp. infected flocks may be a source of these bacteria in the corresponding carcasses, but a cross-contamination during the transportation and slaughter process is also very important.

## 1. Introduction

Campylobacteriosis has been highlighted as the most frequently reported foodborne illness in the European Union, with 246,158 confirmed human cases in 2017, and an incidence rate of 64.8 cases per 100,000 people [1]. The annual number of campylobacteriosis cases in the European Union was estimated at 9 million, which means that only about 2% of all cases are reported [2].

The prevalence of *Campylobacter* spp. in poultry meat is high [1]. Poultry meat is considered to be the main foodborne source of human campylobacteriosis. Campylobacteriosis is often associated with the consumption of undercooked meat, or as a result of handling raw poultry [3].

Epidemiological studies have been carried out to evaluate the possible sources of *Campylobacter* spp. and related risk factors at the farm level. Various factors have been associated with the infection including the flock age, number of days between flocks, number of houses on the farm, thinning, positive status of the previous flock, presence of infected neighbours within 1 to 2 km, presence of rodents, flies, wild animals, domestic animals, personnel, equipment, water, feed, type of ventilation system, and climate related factors, especially the temperature [4,5,6]. The reported prevalence of *Campylobacter* spp. in broiler flocks varies from 42.5% to 100% [7,8,9].

In the European Union the reported prevalence of *Campylobacter* spp. in broiler flocks was 27.3% and 12.3% in 2016 and 2017, respectively [1,7].

Poultry meat may be contaminated with *Campylobacter* during the slaughter process, with major points of cross-contamination on the slaughter line [8]. The reported prevalence of *Campylobacter* in carcasses at the slaughterhouse varies from 34.9% to 100% of the carcasses [4,8,9]. Various risk assessments indicate that reductions of *Campylobacter* counts on broiler carcasses could result in significant decreases in associated human cases [2]. The reductions of 1 to 2 log units in the numbers of *Campylobacter* counts on poultry could be achieved after washing with organic acids, but these treatments are not permitted on poultry in the EU [10,11].

The aim of the study was to investigate the prevalence of *Campylobacter* in birds from three farms at different ages and the corresponding carcasses and poultry products, as well as the effect of certain stages in the poultry slaughter process.

## 2. Materials and Methods

Samples from broiler flocks at the farm level and the corresponding carcasses and poultry meat products were collected between May and June 2014. The slaughterhouse environment and processing equipment were also sampled.

### 2.1. Farms

Three poultry broiler farms (A, B and C) located in the North of Spain were selected. The criteria for the farm selection were the following: similar size, similar capacity, the same veterinarian/consultant and the location in the north of Spain and maximum distance between farms below 100 km. Commercial broiler flocks were from Cobb industrial breeds. Farm A had two houses: AA and AB. House AA had a capacity of 24,500 birds, and House AB was larger, holding 35,500 birds. Farm B had only one house with a capacity of 31,000 birds. Farm C had one house with a capacity of 35,000 birds. Some birds were removed from the farms during the third week of the rearing period, in order to improve animal welfare and to obtain small birds for roasting.

Samples from the same flock at different ages were taken at each farm. The equipment used for the sampling was swabs (Deltalab, Spain). Cloacal swab samples were taken by the farm veterinarian from 7, 14, 35 and 42-day-old animals for a determination of *Campylobacter* spp. Each sampling day, 10 swab samples from 10 different birds were taken and analyzed separately, except on farm A where 10 samples were taken for each house. Swab samples were immediately transported to the laboratory under cooled conditions, and examined for the presence of *Campylobacter* spp. A total of 160 samples from 3 farm broiler flocks were collected (Table 1).

### 2.2. Slaughterhouse and Processing Plant

The same broiler flocks analyzed on the farms were transported to a slaughterhouse at the age of 42 days. No feed and water was given to animals the previous 12–18 h before transportation. The transportation from each farm to the slaughterhouse was carried out in different vehicles. The time for transportation was between 40 and 60 min. The slaughterhouse processes 1,800,000 broilers per month. The slaughterhouse had an automated processing line in which the chickens pass through a gas stunner, neck cutter, bleed out, scalding tanks at 52 °C for 30 s, a plucking machine, an automatic eviscerator, followed by external washing with water at 15 °C, and finally air chilling. The eviscerated carcasses were refrigerated for 2.5 h in a tunnel reaching temperatures of 4–6 °C. After refrigeration, the carcasses were cut in the adjacent further processing plant in order to obtain different poultry meat portions such as wings, breasts, and legs, among others. Since breasts are usually marketed without skin, the skin was removed from this product.

Broilers from the three farms were slaughtered the same day. They were slaughtered at the beginning of the production in order to avoid contamination with previous flocks. Broilers from Farm C, which were *Campylobacter* negative at the farm level, were slaughtered before the *Campylobacter* positive flocks (broilers from Farms A and B). The sampling of the carcasses was performed in the middle of the flock. Between the flocks, a cleaning was carried out.

Broiler carcasses from the three farms were examined at selected stages of slaughter for *Campylobacter* spp.: after defeathering, after evisceration, after washing and after refrigeration. For the carcass sampling, 10 g of skin samples were taken. The samples from the poultry meat were also taken at the further processing plant. The breast, legs and wings were selected for this purpose as they are the most widely consumed parts. Table 2 and Table 3 show the number of carcass samples taken at each process stage and the poultry meat portions, respectively. The samples were transported to the laboratory under cooled conditions.

The slaughterhouse environment and processing equipment were also sampled in order to evaluate the possibility of cross-contamination. Four samples were taken at each sampling time in each sampling point. The samples were also taken from the equipment and the water used in the process. The samples were taken from the scalding water, the defeathering machine, the water used for washing the final carcasses (before refrigeration), and from the work tables at the processing plant. The samples were taken at the beginning of the production when the surfaces and equipment were clean and disinfected, and later in the middle and at the end of the working day (before cleaning and disinfection). The samples were transported to the laboratory under cooled conditions.

Swab samples (Deltalab, Spain) were taken from the transport crates used for transporting birds from Farm C (flock *Campylobacter* negative at the farm level). The samples were taken after unloading the birds (dirty transport crates) (*n* = 4) and after cleaning and disinfection (*n* = 4). The transport crates used followed the animal welfare conditions, with a capacity of 10–12 animals. For transporting 600 animals from Farm C, 50 transport crates were used. After cleaning and disinfection, the crates were visible clean. An automatic washer was used for the cleaning and disinfection of the transport crates.

### 2.3. Campylobacter Determination

At the farm level, samples were obtained from the cloaca of individual birds by using swabs. The presence of *Campylobacter* was determined as follows. Swabs were homogenized for 30 s with 225 mL of Bolton broth (Oxoid, Basingstoke, Hampshire, UK) with a *Campylobacter* selective supplement (Oxoid). The samples were incubated at 42 °C for 24–48 h in a microaerobic atmosphere using the CampyGen kit (Oxoid). The samples were analyzed by the Polymerase Chain Reaction (PCR) method using the Bax system (Oxoid). An analysis was performed according to the manufacturer’s instructions.

A *Campylobacter* enumeration was done on modified charcoal cefaperazone deoxycholate agar (mCCDA) (Oxoid) according to ISO 10272-2 [12]. A confirmation of presumptive colonies was performed according to the ISO 10272-2 principles [12].

In order to analyze the presence of *Campylobacter* in carcasses at the slaughterhouse, 10 g of skin samples were taken at selected stages. The samples were analyzed by the polymerase chain reaction method (PCR method), following the same procedure described at the farm level

In order to determine the presence of *Campylobacter* in the breasts, legs and wings, twenty five grams of skin and muscle (in the same proportion) were aseptically weighed and homogenized in a Stomacher (IUL, Barcelona, Spain) for 2 min with 225 mL of Bolton broth (Oxoid) with a *Campylobacter* selective supplement (Oxoid). The samples were analyzed by the PCR method, following the same procedure as that described for swabs. Since the breasts had no skin, the samples were taken only from muscle.

For the quantification of *Campylobacter* in water, 25 mL was taken, the enumeration was done on modified charcoal cefaperazone deoxycholate agar (mCCDA) (Oxoid) according to ISO 10272-2 [10]. In order to analyze the presence of *Campylobacter* spp., the samples were also analyzed by the PCR method described above.

Swab samples were also taken for the quantification of *Campylobacter* in transport crates and equipment surfaces. An enumeration was performed on modified charcoal cefaperazone deoxycholate agar (mCCDA) (Oxoid) according to ISO 10272-2 [12]. In order to analyze the presence of *Campylobacter* spp., samples were also analyzed by the PCR method described above.

### 2.4. Statistical Analysis

An analysis of variance was performed using the SYSTAT program for Windows; Statistics version 5.0 (Systat Software, Inc., Evanston, IL, USA). Tukey’s test for the comparison of means was performed using the same program. The plate count data were converted to logarithms prior to their statistical treatment. The significance level was defined at *p* < 0.05.

## 3. Results

### 3.1. Campylobacter Presence at Farm Level

Table 1 shows the *Campylobacter* spp. prevalence in cloaca in chicken from the 3 farms studied following the PCR method. *Campylobacter* spp. was not detected in 7 and 14-day-old broilers in any of the three farms. However, *Campylobacter* was detected in Farms A and B in 35 and 42-day-old broilers. *Campylobacter* was not detected in any birds from Farm C. High levels of *Campylobacter* (over 6 log CFU/mL) were detected in Farms A and B in 35-day-old animals. Two houses (AA and AB) were analyzed in Farm A, and both were found to be *Campylobacter* positive on day 35. *Campylobacter* counts were above 6 log CFU/mL on day 35. On day 42, *Campylobacter* spp. counts were 5.64 ± 0.25 log CFU/mL and 4.79 ± 0.35 log CFU/mL in houses AA and AB, respectively, and 4.12 ± 0.40 log CFU/mL in farm B. A decline of *Campylobacter* counts was observed on day 42 compared to samples taken on day 35.

Significant differences (*p* < 0.05) in *Campylobacter* counts in cloaca swabs were found between the 7 and 14-day-old and the 35 and 42-day-old broilers in Farms A and B. Significant differences (*p* < 0.05) were also found between older birds (over 35 days old) from Farms A or B and from Farm C. Significant differences (*p* < 0.05) were also found between the 35 and 42-day-old broilers in Farms A and B.

### 3.2. Campylobacter Presence at Slaughterhouse and Processing Plant

Table 2 shows the prevalence of *Campylobacter* spp. in carcasses from 3 farms at different stages of processing. After defeathering, *Campylobacter* spp. was detected in all of the carcasses from Farm A. After evisceration, a decrease was observed with 80% of positive samples. After chilling, *Campylobacter* spp. was detected in 90% of the carcasses. In the processing plant it was detected in 73.33% of the samples.

*Campylobacter* spp. was detected in all the carcasses from Farm B after defeathering, evisceration and after refrigeration. After washing, a decrease was observed with 60% of the samples showing positive. In the processing plant *Campylobacter* spp. was detected in all of the samples. *Campylobacter* spp. was detected in all of the carcasses from Farm C.

Table 3 shows the prevalence of *Campylobacter* in poultry meat portions from the 3 farms at the processing plant following the PCR method. In products from Farm A, *Campylobacter* spp. was detected in 60%, 100% and 60% of the legs, breast and wings, respectively. *Campylobacter* spp. was detected in all of the poultry meat portions analyzed from Farm B. All of the meat samples (legs, breasts and wings) from Farm C were *Campylobacter* positive.

*Campylobacter* was detected in transport crates after unloading the birds (dirty transport crates) with counts of 3.76 ± 0.12 log CFU/cm^2^, ranging from 3.60 to 3.90 log CFU/cm^2^. *Campylobacter* was also detected in transport crates after cleaning and disinfection with counts of 2.54 ± 0.8 log CFU/cm^2^, ranging from 1.30 to 3.48 log CFU/cm^2^.

All the equipment and environment samples analyzed by the PCR method were *Campylobacter* negative at the beginning of the working day (after cleaning and disinfection). However, at the middle and the end of the working day, *Campylobacter* spp. was detected in the scalding water and the defeathering machine. *Campylobacter* was only detected on the working table at the end of the working day. Table 4 shows the *Campylobacter* spp. counts in the slaughterhouse environment at different times during the working day following the ISO 10272-2 method.

## 4. Discussion

In our research *Campylobacter* was not detected in any birds under 2 weeks of age from two of the three farms that became colonized with *Campylobacter* spp. during the growing period (Farms A and B). *Campylobacter* was first detected in these farms after 35 days. Other authors have also reported that *Campylobacter* is rarely isolated from broiler flocks until the birds are at least 2 or 4 weeks old [13]. This fact could be explained since vertical transmission from parent flocks is not considered a major source of colonization [13], and because of the protective action of maternal antibodies in young birds [14].

According to the current study, the age of broilers could be associated with an increased risk of *Campylobacter* in poultry flocks. This has also been suggested by other authors [15]. A longer exposure to possible sources of infection could explain the higher probability of infection when the bird age increases [16].

The primary sites of colonization of *Campylobacter* in poultry are the caeca, colon and cloaca [3]. Infected birds carry a very high *Campylobacter* load in their gastrointestinal tract. In the current study, high levels of *Campylobacter* were detected in cloacal samples in positive flocks. A decline of *Campylobacter* spp. numbers was observed on day 42 compared to samples taken on day 35. These results are in agreement with those reported by Achen et al. [17], who observed that once the birds are colonized by *Campylobacter*, the highest levels are reached after 5 days. A slight decline of colonization occurs after about 4 weeks, and negative birds may then occur. However, other authors have reported that the level of *Campylobacter* did not change significantly as the flock continued to grow [15]. Other authors have also observed that, after infection, birds rapidly exhibit high levels of *Campylobacter* in the large intestine, caecum and cloaca, with counts of 5–9 log CFU/g commonly being observed [3,18].

Another factor associated with an increased risk of *Campylobacter* in poultry flocks is the number of houses on the farm [19]. In the present study, Farm A had two houses, and the presence of *Campylobacter* was detected in birds from both houses after day 35. According to Lyngstad et al. [20], having more than one broiler house on the farm could be important, since when one house is infected the disease could be spread to other houses on the farm by biological vectors, humans and equipment, depending on farm biosecurity measures.

In the current study, the farms carried out a practice known as thinning, involving the partial depopulation of the broiler houses. As has been reported by other authors, thinning causes an increased risk of introducing *Campylobacter* into a flock [4,9]. Thinning methods may vary from country to country, which could explain the differences observed in the *Campylobacter* prevalence [21]. However, other authors have not found any correlation between thinning and *Campylobacter* flock positivity, considering the broiler age as the most probable factor increasing the risk [22]. Thinning was carried out in the third week of the rearing period (by day 21), and all the *Campylobacter* positive birds were at least 35 days old.

Since *Campylobacter* flock positivity increases with age and after thinning, it would be useful to check whether a flock is *Campylobacter* positive or negative as close as possible to slaughter time. If not, those flocks that become positive between sampling and slaughter will not be detected. In our research, birds were analyzed for the presence of *Campylobacter* spp. on the same day they were transported to the slaughterhouse for processing (day 42). Birds from one of the three farms (Farm C) were *Campylobaacter* negative before being taken to the slaughterhouse.

Our results show that the prevalence of *Campylobacter* increases when the bird age increases. In order to compare the prevalence reported for different studies, the bird’s age should be taken into account.

The different levels of *prevalence* of *Campylobacter* positive flocks could be due to climatic conditions. Nylen et al. [23] reported that the *Campylobacter* prevalence varies according to the season of rearing, and is higher in the summer than in the winter or spring. According to Nylen et al. [23], these differences could reflect levels of environmental contamination, given that poultry houses have more ventilation in the summer, thus increasing the contact with the outside environment. In the present study, samples were taken in the late spring (May and June), which could explain the higher prevalence compared to other studies, with two of the three farms studied showing *Campylobacter* bird colonization on day 35 of the rearing period.

The external environment around the broiler farm could be an important source of *Campylobacter* spp. This pathogen can be present in wild and domestic animals. Domestic animals are often *Campylobacter* positive, and their access to poultry flocks should not be allowed. *Campylobacter* spp. has been isolated in flies sampled on chicken farms, and these can be a source for these bacteria. Since the cross contamination between successive flocks on broiler farms can occur, it is essential that farm cleaning and disinfection was effective [13,24,25].

Contaminated water and feed can also introduce *Campylobacter* spp. into poultry flocks. It is important that birds receive water that is of potable quality [13,24].

Biosecurity measures on the farm can protect against *Campylobacter* infection. The importance of hygiene and biosecurity measures in the prevention of a *Campylobacter* infection has been pointed out by several authors [26]. However, Vidal et al. [27] observed that although biosecurity measures were addressed, the levels of *Campylobacter* spp. contamination remained high. According to some studies, *Campylobacter* species on the carcasses originate mainly from the gastrointestinal tracts of live birds [28]. This means that the control of *Campylobacter* in poultry at the farm level would reduce the risk of human exposure to this pathogen and have a significant impact on food safety. The reduction of environmental exposure through biosecurity measures would be of great interest [29].

In this study all the samples taken from birds from Farm C at the slaughterhouse and further processing plant were *Campylobacter* positive, although the original flock was negative. These results suggest that the poultry carcasses were cross-contaminated during the transportation and slaughter process. The crates used to transport birds to the slaughterhouse could have been the source of contamination for the birds from Farm C. *Campylobacter* was isolated in the transport crates, even after cleaning and disinfection. Additionally, Ellerboroek et al. [30] found *Campylobacter* positive samples in the transport crates before and after cleaning and disinfection. Inadequate cleaning and disinfection of the transport crates could therefore be a source of contamination of *Campylobacter* negative flocks [31]. Other authors have detected *Campylobacter* in transport crates before bird loading and have found evidence to suggest that the colonization of broiler flocks could occur during the transport [31].

Our results confirm those reported by Allen et al. [32], who found a cross-contamination of carcasses from *Campylobacter*-negative flocks, even when they were processed in the slaughterhouse after *Campylobacter* negative birds. Others authors also found carcasses contaminated with *Campylobacter*, even when the bacteria were not isolated from the chickens upon arrival at the abattoir [7]. The contamination of broiler carcasses during the processing may occur at various points, such as during the scalding, plucking, defeathering, evisceration or chilling operations [18,32]. Moreover, *Campylobacter* can survive on the surface of slaughterhouse equipment despite cleaning and sanitizing and may contaminate carcasses during the slaughter process [33].

On the other hand, some poultry samples were negative for *Campylobacter* even though the original flocks were *Campylobacter* positive before slaughter (Farms A and B). In contrast, Seliwiorstow et al. [34] observed that all of the caecal samples collected at the slaughterhouse from *Campylobacter* colonized flocks were positive for *Campylobacter*. Furthermore, Gruntar et al. [35] isolated *C. jejuni* in all of the faecal samples taken at an abattoir from positive birds. In this study, cloacal samples taken after defeathering in carcasses from Farms A, B and C were *Campylobacter* positive.

In our study all the samples taken from the scalding water, the defeathering machine, the washing water and the work table were *Campylobacter* negative at the beginning of the working day (after cleaning and disinfection). At the end of the working day (after slaughtering), *Campylobacter* was detected in the scalding water, the defeathering machine and the working table. Gruntar et al. [35] also reported that all of the slaughterhouse environment samples were *Campylobacter* negative before slaughtering and positive after slaughtering. However, these authors observed one exception, the scalding water, which remained negative after slaughtering. In contrast, Kudirkiene et al. [36] indicated that *Campylobacter* can remain in the slaughterhouse environment after disinfection, and is a potential source of poultry meat contamination. Different results found in the bibliography could be explained because some slaughterhouses control *Campylobacter* contamination better than others [34].

It is important to remember that broilers are often colonized by large numbers of *Campylobacter* without the animal showing any clinical signs. Colonized birds enter the slaughterhouse with high numbers of *Campylobacter* in their intestinal tract as well as on their feathers and skin. Thus, *Campylobacter* can be found throughout the slaughtering process. In consequence, the contamination of work surfaces, equipment, water and air could occur [34]. Since there is a high risk that campylobacters present on infected birds will be transmitted to other carcasses being processed, it is important to minimize the carcass contamination during slaughtering and further processing. Preventive measures include temperature controls (scalding water, washing water, carcasses, poultry products), chemical interventions, water replacements, chlorinated-water sprays for equipment and working surfaces, as well as adequate cleaning and disinfection of equipment and contact surfaces. [10,11,24].

Wieczorek and Osek [37] observed that some carcasses were contaminated with *Campylobacter* although the flocks were *Campylobacter* negative. On the other hand, some carcasses were negative for *Campylobacter* although the original flocks were *Campylobacter* positive. This fact could be explained by internal contamination during the slaughter of the broilers. A cross-contamination to the flocks processed the following day, after the plant has been cleaned and disinfected, has also been reported by Genigoergis et al. [38]. The modern-day slaughter of poultry is a highly automated process. Since healthy broilers are often carriers of *Campylobacter* spp., carcasses might become contaminated during the slaughter.

*Campylobacter* spp. does not replicate in food, but since a relatively low dose is sufficient to cause an infection [3], it is important to reduce *Campylobacter* counts in poultry meat. A decrease in both the level of prevalence of *Campylobacter* in flocks and the load of bacteria in contaminated carcasses would help decrease the number of human cases [27].

Poultry meat may be contaminated with *Campylobacter* during the slaughter processing. In this study, all of the carcasses analyzed after defeathering were *Campylobacter* positive, even in the carcasses from *Campylocater* negative farms. This high prevalence could be explained by the previous stage, scalding [36]. According to Humphrey [39], the scalding water is the main source of cross-contamination on broiler carcasses, and contaminates both the surface and muscle tissue of the carcasses. In contrast, other authors have reported that scalding could reduce *Campylobacter* contamination on carcasses [40]. In the current study, the scalding water temperature was 52 °C; this temperature could have a limited effect on *Campylobacter* reduction, as shown by Yang et al. [41]. Higher scalding temperatures seem favorable in terms of reducing *Campylobacter* counts, but lesions to the broiler skin can affect the quality of fresh poultry meat. In our research, *Campylobacter* was not detected in scalding water at the beginning of the working day (after cleaning and disinfection), but it was found after slaughtering (samples taken at the middle and end of the working day) with counts ranging between 3.3 and 4.9 log CFU/mL. These results confirm those reported by Lebner et al. [34] who found that scalding water was *Campylobacter* positive in all of the samples ranging between 2.2 and 3.7 log CFU/mL. They used scalding water at temperatures ranging between 53 and 53.9 °C, slightly higher than in our study (52 °C). Since *Campylobacter* spp. was found in scalding water during the processing, this should be considered a critical step for possible *Campylobacter* spp. cross-contamination between carcasses [42].

Defeathering could increase the *Campylobacter* carcass contamination due to the presence of fecal material in the feather-picking machine [43]. In our current research, *Campylobacter* was not detected in the defeathering machines at the beginning of the working day (after cleaning and disinfection), but it was found after slaughtering (samples taken at the middle and end of the working day). Peyrat et al. [27] also detected *Campylobacter* in samples taken from defeathering machines before cleaning, and even after they had been cleaned (14% positive samples).

Evisceration is also an important contamination point due to possible intestinal ruptures during the mechanical removal of the intestines. Small amounts of caecal contents (5 mg) can increase the numbers of *Campylobacter* on eviscerated broiler carcasses [44] because the bacteria are found abundantly in the large intestine, cloaca and caeca, where 5 to 9 log CFU/g have been observed [3]. However, some studies have reported a *Campylobacter* reduction after evisceration, probably due to the washing of the carcasses. In the present study a reduction in *Campylobacter* positive samples was observed after washing in carcasses from Farm B. According to Seliwiorstow et al. [45], in order to decrease contamination, evisceration machines should be correctly adjusted to the size of the processed birds. These authors associated the percentage of ruptured gastrointestinal packages and damaged cloaca with a higher *Campylobacter* count in carcasses.

In the present study, chilling did not reduce the prevalence of *Campylobacter* in carcasses. Similar results were reported by Ellerbroek et al. [30], as a higher prevalence of *Campylobacter* on carcasses was observed after chilling (96.7%) compared to after scalding (91.1%). In contrast, other authors reported that chilling tends to reduce the *Campylobacter* prevalence [40].

According to Bare et al. [46], *Campylobacter* is distributed over the whole broiler carcass, although there are variations between skin sites. In our research the highest number of *Campylobacter* positive samples was observed in the breasts, in which all of the samples were positive. All of the meat samples (legs, breasts and wings) from Farm C were *Campylobacter* positive. The increase in the *Campylobacter* prevalence after chilling due to cross-contamination with water was reported by Smith et al. [47], who observed that the presence of *Campylobacter* increased from 79% to 100% after chilling. The cross-contamination of broiler carcasses could occur during chilling from equipment or air [48].

In the present study a decrease in the prevalence of *Campylobacter* in the wings and legs from Farm A was observed compared to samples taken after chilling. The behavior was different depending on the product and the farm. The cross-contamination during the portioning could be associated to the presence of contaminated equipment, work surfaces or workers’ hands [49].

Our results are in agreement with those reported by Hue et al. [50] and Powell et al. [9], who observed that the prevalence of *Campylobacter* was higher on carcasses than in caeca. These findings support the idea that cross-contamination may occur during the slaughter and further processing. The presence of contaminated equipment, work surfaces, process water and air increases the probability of carcass contamination.

## 5. Conclusions

In conclusion, the present study shows that *Campylobacter*-infected flocks may be a source of these bacteria for the corresponding carcasses, but cross-contamination during the transportation and slaughter process is also of great importance. During the processing, the spread of *Campylobacter* and the cross-contamination of broiler carcasses by the bacteria present in the intestinal content may cause hygiene problems. Our results suggest that a control at the slaughterhouse is important, as are the measures taken at the farm level and during transportation. Hygiene measures on the farm, during transportation and in slaughterhouse are crucial for the reduction of *Campylobacter* spp.

## Figures and Tables

**Table 1:** Prevalence of *Campylobacter* spp. in broilers from three farms: A, B and C.

Farm	House	Broiler Age	Number of Samples	Number of Positive Samples	Prevalence of *Campylobacter* spp. (%)
A	A	7 days	10	0	0
	B	7 days	10	0	0
	A	14 days	10	0	0
	B	14 days	10	0	0
	A	35 days	10	10	100
	B	35 days	10	10	100
	A	42 days	10	10	100
	B	42 days	10	10	100
B	A	7 days	10	0	0
	A	14 days	10	0	0
	A	35 days	10	10	100
	A	42 days	10	10	100
C	A	7 days	10	0	0
	A	14 days	10	0	0
	A	35 days	10	0	0
	A	42 days	10	0	0

**Table 2:** Prevalence of *Campylobacter* spp. in carcasses from the 3 farms at different stages of processing.

Farm	Processing Stage	Number of Samples	Number of Positive Samples	Prevalence of *Campylobacter* spp. (%)
A	After defeathering	10	10	100.00
	After evisceration	15	12	80.00
	After washing	10	9	90.00
	After refrigeration	10	9	90.00
	Processing plant	30	22	73.33
B	After defeathering	10	10	100.00
	After evisceration	10	10	100.00
	After washing	10	6	60.00
	After refrigeration	10	10	100.00
	Processing plant	30	30	100.00
C	After defeathering	10	10	100.00
	After evisceration	10	10	100.00
	After washing	10	10	100.00
	After refrigeration	10	10	100.00
	Processing plant	30	30	100.00

**Table 3:** Prevalence of *Campylobacter* spp. in poultry meat portions from the 3 farms at the processing plant (%).

Farm	Portion	Number of Samples	Number of Positive Samples	Prevalence of *Campylobacter* spp. (%)
A	Legs	10	6	60.00
	Breast	10	10	100
	Wings	10	6	60.00
B	Legs	10	10	100
	Wings	10	10	100
	Breast	10	10	100
C	Legs	10	10	100
	Breast	10	10	100
	Wings	10	10	100
	Total	90	82	

**Table 4:** *Campylobacter* spp. counts in the slaughterhouse environment at different times during the working day: equipment (log CFU/cm^2^) and water (log CFU/mL) (*n* = 4).

Sample	Initial	Middle	End
Scalding water (52 °C)	ND	4.22 ± 0.56 ^1^	3.92 ± 0.59
Defeathering machine	ND	2.79 ± 0.31	2.35 ± 0.65
Washing water (15 °C)	ND	ND	ND
Working table	ND	ND	1.23 ± 0.13

ND, not detected; ^1^ Mean ± standard deviation.
